# Effect of Chain-Extenders on the Properties and Hydrolytic Degradation Behavior of the Poly(lactide)/Poly(butylene adipate-*co*-terephthalate) Blends

**DOI:** 10.3390/ijms141020189

**Published:** 2013-10-10

**Authors:** Weifu Dong, Benshu Zou, Yangyang Yan, Piming Ma, Mingqing Chen

**Affiliations:** The Key Laboratory of Food Colloids and Biotechnology, Ministry of Education, School of Chemical and Material Engineering, Jiangnan University, 1800 Lihu Road, Wuxi 214122, China; E-Mails: wfdong@jiangnan.edu.cn (W.D.); zoubenshu_2008@126.com (B.Z.); yangyang8461@126.com (Y.Y.)

**Keywords:** PLA, PBAT, compatibilization, properties, degradation

## Abstract

Biodegradable poly(lactide)/poly(butylene adipate-*co*-terephthalate) (PLA/PBAT) blends were prepared by reactive blending in the presence of chain-extenders. Two chain-extenders with multi-epoxy groups were studied. The effect of chain-extenders on the morphology, mechanical properties, thermal behavior, and hydrolytic degradation of the blends was investigated. The compatibility between the PLA and PBAT was significantly improved by *in situ* formation of PLA-*co*-PBAT copolymers in the presence of the chain-extenders, results in an enhanced ductility of the blends, e.g., the elongation at break was increased to 500% without any decrease in the tensile strength. The differential scanning calorimeter (DSC) results reveal that cold crystallization of PLA was enhanced due to heterogeneous nucleation effect of the *in situ* compatibilized PBAT domains. As known before, PLA is sensitive to hydrolysis and in the presence of PBAT and the chain-extenders, the hydrolytic degradation of the blend was evident. A three-stage hydrolysis mechanism for the system is proposed based on a study of weight loss and molecular weight reduction of the samples and the pH variation of the degradation medium.

## 1. Introduction

Polymeric materials derived from biomass have received great attention in recent years because of limited petroleum resources and the environmental concerns [1,2]. Poly(lactide) (PLA) is one of the most extensively studied bio-based and biocompostable aliphatic polyesters [3–5]. Several favorable properties, such as high strength and stiffness at room temperature, make it promising as a substitute for conventional petroleum-based polymers. However, the application of PLA is still limited due to its high price, low heat distortion temperature, and brittleness [6,7].

The mechanical properties, notably the brittleness, of PLA can be improved by either copolymerization [8,9] or blending [10–23]. Compared with copolymerization, blending is more economical and convenient, therefore, traditional (co-)polymers such as poly(urethane) (PU) [10], poly(ethylene) (PE) [11], poly(ethylene-co-glycidyl methacrylate) (EGMA) [12], poly(ethylene-co-acrylate) (TPO) [13], ethylene-co-vinyl acetate (EVA) [14] and poly(ethylene-*co*-octene) (POE) [15] are used to improve the toughness of the PLA. As a result, tough PLA were obtained via such modification. On the other hand, PLA was blended with ductile modifiers such as poly(hydroxyalkanoate)s (PHAs) [16], poly(caprolactone) (PCL) [17–19], poly(butylene succinate) (PBS) [20,21] and poly(butylene adipate-*co*-terephthalate) (PBAT) [22–26] to make fully bio-based and/or biocompostable materials. Among these modifiers PBAT is a petroleum-based but fully biocompostable copolymer with high ductility. PBAT was initially designed by BASF, also known “Ecoflex^®^”, which was used world-wide and studied for packaging applications. In recent years PBAT, developed in China by e.g., Xinfu Pharmaceutical Co. Ltd., has received considerable interest. The elongation at break of PLA was increased from 4% to 200% by addition of 20 wt % of the PBAT (Ecoflex^®^) [22]. However PLA showed complete phase separation with PBAT leading to a limited improvement in the toughness by physical blending.

To obtain high toughness of PLA blends, compatibilization is essential which can be performed by addition of pre-made copolymers or by an *in situ* reaction between the polymers during blending. In PLA/PCL [19] and PLA/PBS [20] blends free-radical initiator, *i.e.*, dicumyl peroxide (DCP) was introduced into the melt to induce in-situ compatibilization. Consequently the particle size of dispersed PCL and PBS reduced significantly accompanied by an increase in interfacial adhesion due to an *in situ* compatibilization, resulting in an improved toughness of the PLA/PCL and PLA/PBS blends. *In situ* compatibilization can also be created in polyester blends via chain-extenders with multi-functional groups such as epoxy, however, few efforts were dedicated to this approach. The PLA and PBAT (Ecoflex^®^) was compatibilized by using glycidyl methacrylate (GMA) but the elongation at break of the compatibilized PLA/PBAT blends was even lower than 10% [25]. The thermal stability of PLA, PBAT (Ecoflex^®^) and PLA/PBAT blends was increased by a chain-extender, *i.e.*, functionalized epoxy (Joncryl ADR-4368), while the elongation at break of the blends was increased maximally to 135% [27]. Moreover, the effect of chain-extenders on the hydrolytic degradation behavior, which is an important feature of the PLA/PBAT blend as a biodegradable material, is not clear yet.

The prime objective of this paper is to provide a simple but effective and economic route to prepare tough biodegradable PLA materials based on reactive (melt-)blending of PLA with PBAT in the presence of chain-extenders. The phase morphology, mechanical properties, thermal properties, and hydrolytic degradation of the PLA and the PLA/PBAT blends with and without chain-extenders are studied in detail.

## 2. Results and Discussion

### 2.1. Compatibilization Effect of the Chain-Extenders on the PLA/PBAT Blends

The brittleness of PLA can be improved by blending with PBAT, however their compatibility is poor due to their immiscibility [22]. To improve the compatibility, two chain-extenders with epoxy groups are applied in this work. The two chain-extenders are Joncryl ADR-4370S, abbreviated as ADR, and 1,6-hexanediol diglycidyl ether, abbreviated as HDE in this paper.

**Figure 1:**
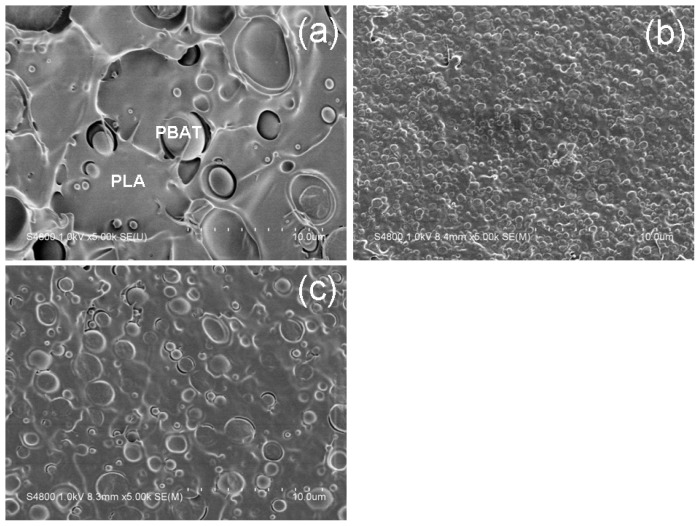
Scanning electron microscopy (SEM) images of the cryo-fractured surfaces of (**a**) PLA/PBAT (80/20, *w*/*w*); (**b**) PLA/PBAT/ADR (80/20/1, *w*/*w*); and (**c**) PLA/PBAT/HDE (80/20/1, *w*/*w*). The domains in the images represent the PBAT while the matrix corresponds to the PLA phase, as indicated in image (**a**).

The effect of chain-extenders on the phase morphology of the PLA/PBAT (80/20, *w*/*w*) blends is shown in Figure 1. It can be seen from Figure 1a, twenty percent of the PBAT is dispersed non-uniformly in the PLA matrix with large domain size (1~5 μm), while adhesion between the PLA and PBAT phases is poor as evidenced by interfacial de-bonding and oval cavities left by the PBAT domains after cryo-fracture. The dispersion of the PBAT domains becomes uniform and the average PBAT domain size is reduced from approximately 3 μm in the PLA/PBAT (80/20, *w*/*w*) blend to approximately 0.5 and 1 μm after addition of 1 wt % of ADR and HDE, respectively. Meanwhile the interfacial adhesion between the PLA and PBAT phases is improved. These results indicate that the compatibility between the PLA and PBAT is greatly enhanced by the incorporation of chain-extenders, which reasonably affects the properties of the blends, see discussion below.

### 2.2. Compatibilization Mechanism of the Chain-Extenders

**Scheme 1:**
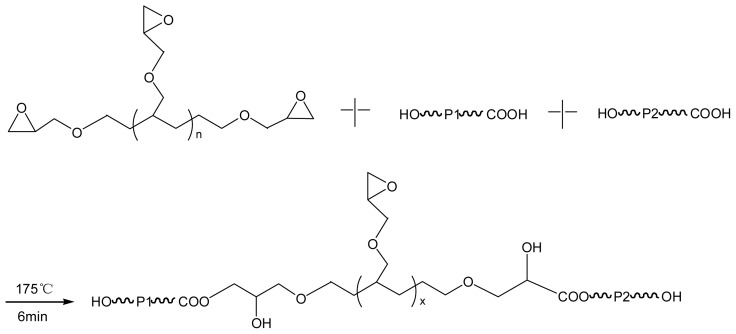
Proposed reactions between the polyesters and the chain-extenders. P1 and P2 represent PLA and/or PBAT chains. X represents reacted polymer chains (P1 and/or P2) and the remained epoxy groups. *n* = 0 or 7 corresponds to the HDE or ADR respectively.

It is known that epoxy groups are reactive with −COOH and −OH groups [25–27]. The chain-extenders used in this work, ADR and HDE, contain nine and two epoxy groups respectively per molecule, which would react with the end groups, *i.e.*, −COOH and −OH, of the PLA and PBAT chains during melt processing. Consequently two or even more macromolecules are combined together via one molecule of chain-extender, as schematically shown in the Scheme 1.

If P1 and P2 as presented in Scheme 1 are the same type of polymer then extended or branched homo-polymers are generated which happens in the PLA matrix and inside of the PBAT domains, while if P1 and P2 are different types of polymers then block copolymers or graft copolymers are formed which occurs at the interface of the blends. The *in situ* formed copolymers (*i.e.*, PLA-*b*-PBAT or PLA-*g*-PBAT) at the interface are responsible for the enhanced compatibility between PLA and PBAT phases [20]. Besides chain-extension, some uncontrollable side reactions such as thermal degradation/hydrolysis, esterification and *trans-*esterification may occur as well.

The chain-extending effect of the ADR and HDE is confirmed by molecular weight (*M*_w_) analyses, as shown in Table 1. PLA is easily degradable upon processing, leading to lower *M*_w_[28]. For comparison all examined samples underwent similar thermal processing. It is found that the number average molecular weight (*M̄*_n_ ), weight average molecular weight (*M̄*_w_ ) and the molecular weight distribution (DPI) of the PLA/PBAT (80/20, *w*/*w*) blend are increased after addition of the ADR or HDE due to their chain-extension effect. Furthermore, ADR is more effective as a chain-extender in comparison with HDE in terms of *M*_w_ probably due to its higher epoxy content. It has to be remarked that a very small amount of non-soluble fraction was observed in the solution of ADR modified samples indicating a branching or even crosslinking structures. It can be interpreted that only part of epoxy groups were reacted since increase in the *M*_w_ is not proportional to the content of the epoxy groups.

### 2.3. Effect of Chain-Extenders on the Mechanical Properties of the PLA/PBAT Blends

**Figure 2:**
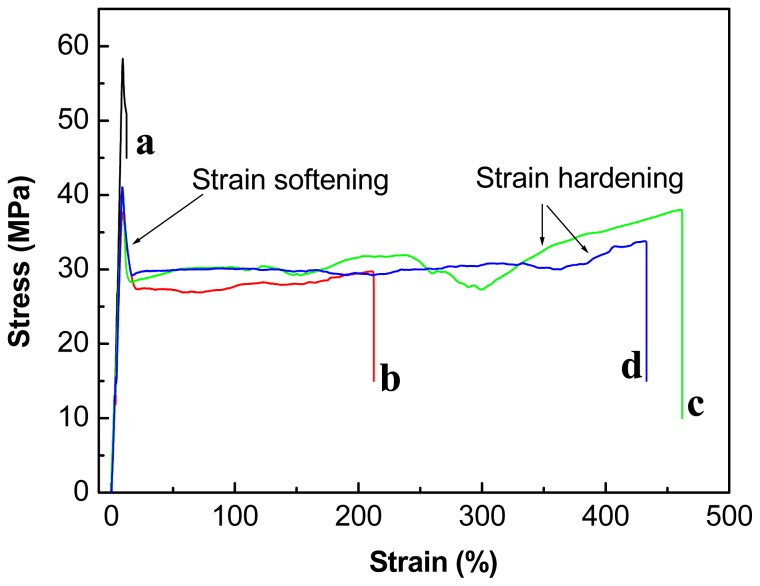
Stress–strain curves of (**a**) PLA; (**b**) PLA/PBAT (80/20, *w*/*w*); (**c**) PLA/PBAT/ADR (80/20/1, *w*/*w*); and (**d**) PLA/PBAT/HDE (80/20/1, *w*/*w*).

The effect of PBAT and two different types of chain-extenders on the mechanical properties of the PLA and the PLA/PBAT blends, respectively, were studied by analyzing the stress-strain behavior, as shown in Figure 2. Neat PLA shows a brittle fracture with a yield stress (σ_y_) of approximately 60 MPa and an elongation at break (ɛ_b_) of approximately 10%. Although the σ_y_ of the PLA was decreased by addition of the PBAT the PLA/PBAT (80/20, *w*/*w*) blend shows distinct yielding and stable neck growth upon drawing, indicating a ductile behavior. Interestingly, the ductility of the PLA/PBAT blends can be greatly improved by incorporation of a small amount of chain-extenders, as shown in Figure 2. The ɛ_b_ of the PLA/PBAT (80/20, *w*/*w*) blend is doubled (*i.e.*, from 210% to approximately 450%) by addition of 1 wt % ADR or HDE, accompanied by a slight increase in the σ_y_. The PLA/PBAT blend with the chain-extenders shows more distinct strain hardening indicating a better delocalization of the strain during tensile testing, thus toughness.

**Figure 3:**
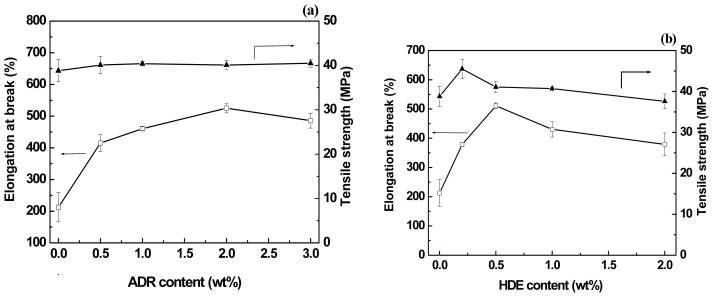
Tensile properties of the PLA/PBAT blends as a function of (**a**) ADR content and (**b**) HDE content.

The tensile properties of the PLA/PBAT (80/20, *w*/*w*) blends with different content of chain-extenders were studied, as shown in Figure 3. It can be seen from Figure 3a that ɛ_b_ of the PLA/PBAT blend increases obviously with the ADR content up to 2 wt %, while the σ_y_ keeps constant. On the other hand, the ɛ_b_ and the σ_y_ of the blend reached to a maximum value at 0.5 and 0.25 wt % of the HDE respectively, as shown in Figure 3b.

Apparently, the ductility and toughness of the PLA/PBAT blend can be improved by a small amount of chain-extenders without compromising on the yield strength. The tensile energy of the PLA/PBAT blends is mainly dissipated during the continuous growth of the neck accompanied by a considerable stress-whitening volume. The stress whitening, in general, is a consequence of numerous cavities and/or crazes formed during deformation. In rubber-toughened polymer system internal rubber cavitation and interfacial de-bonding can be distinguished. In literature, interfacial de-bonding for PLA/PBAT blends was proposed as toughening mechanism [22]. As the compatibility and interfacial adhesion between the PLA and the PBAT phases were improved, the stress and strain can be transferred more efficient from the PLA matrix to the PBAT domains after addition of the chain-extenders [29]. Meanwhile, the number of PBAT domains is increased due to the improved compatibility, as shown in Figure 1, resulting in a significant reduction of inter-particle distance. Consequently the ductility of the PLA/PBAT blends is improved [30].

### 2.4. Effect of Chain-Extenders on the Thermal Behavior of the PLA/PBAT Blends

**Figure 4:**
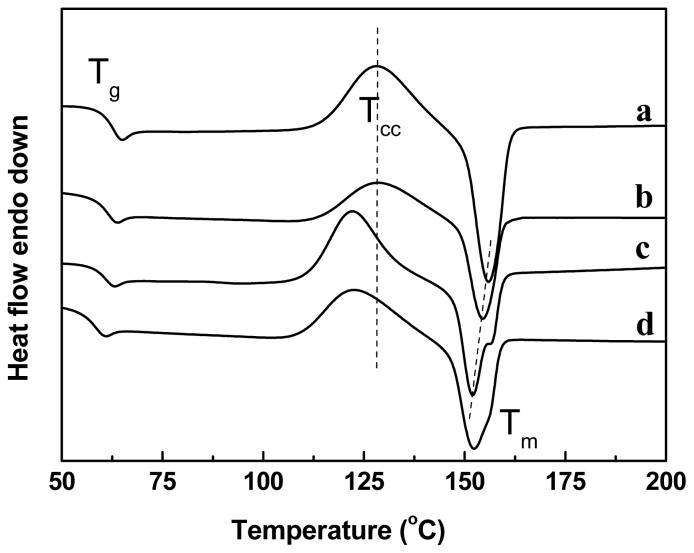
The second heating DSC curves of PLA and PLA/PBAT blends: (**a**) PLA; (**b**) PLA/PBAT (80/20, *w*/*w*); (**c**) PLA/PBAT/ADR (80/20/1, *w*/*w*); and (**d**) PLA/PBAT/HDE (80/20/1, *w*/*w*).

The thermal properties of the PLA and PLA/PBAT blends with and without chain-extenders are studied by using differential scanning calorimeter (DSC). The second heating run curves of the samples are depicted in Figure 4, and the thermal parameters, such as glass transition temperature (*T*_g_), cold crystallization temperature (*T*_cc_), melting temperature (*T*_m_), and melt enthalpy (*ΔH*_m_), are listed in Table 2. Neat PLA has *T*_g_, *T*_cc_, and *T*_m_ of 65, 128, and 156 °C respectively. The cold crystallization of the PLA in the blend is accelerated after addition of the chain-extenders as evidenced by a decrease in *T*_cc_ (by 7 °C) with narrow crystallization peaks and an increase in the *ΔH*_m_. Thus, the large number of PBAT domains (small in size) show a heterogeneous nucleation effect on the PLA.

The *T*_m_ of PLA in the blend is suppressed after addition of the chain-extenders, as shown in Figure 4, which may be due to the less perfection of the PLA crystals and a reduction in the lamellar thickness resulted from the branching or even crosslinking (in the case of ADR) structures in the presence of chain-extenders.

Meanwhile, double-melting peaks are observed which is typical for polyesters and can be explained by a melt/re-crystallization/re-melt mechanism [31]. It was reported that the crystals melted at different temperatures had similar structure but different lamellar thickness [22]. The melting of the PBAT in the blend was not detected probably due to (1) the crystallization of PBAT domains was restricted due to a confinement effect or (2) the small melting peak (*T*_m-PBAT_ = 112 °C) was masked by the cold crystallization peaks of the PLA phase.

### 2.5. Hydrolytic Degradation Behavior of the PLA/PBAT Blends

The hydrolytic degradation rate of polyesters such as PLA is affected by the hydrolytic medium, temperature and molecular structures *etc.* [32–34]. The hydrolytic degradation behavior of the PLA, PBAT and PLA/PBAT blends are studied by means of morphological analysis, weight loss, molecular weight measurement, and pH variation of the solution. To be followed in a reasonable time range the hydrolytic degradation experiments were performed initially in an aqueous alkali at 60 °C.

#### 2.5.1. Morphological Analysis and Weight Loss

**Figure 5:**
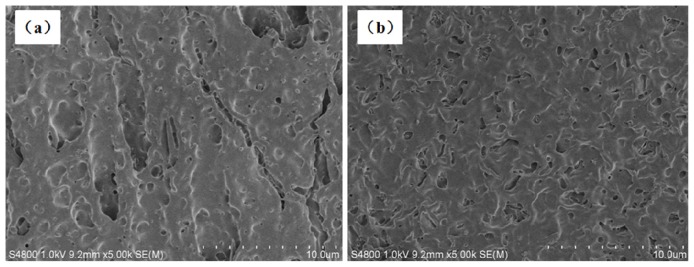
SEM images of the sample surfaces after eight-week hydrolytic degradation showing the erosion phenomena: (**a**) PLA and (**b**) PLA/PBAT (80/20, *w*/*w*).

Transparent PLA samples gradually turned to be white in the initial four weeks of the degradation. The PLA and PLA/PBAT samples became fragile and even lose integrity after four weeks while neat PBAT started to be fragile after eight weeks, indicating a better resistance of the PBAT to the hydrolysis. The erosion of the samples due to hydrolysis was observed by using SEM, as shown in Figure 5. The pothole and cracks in the samples are attributed to the dissolution of the oligomers that are formed by the hydrolysis process.

**Figure 6:**
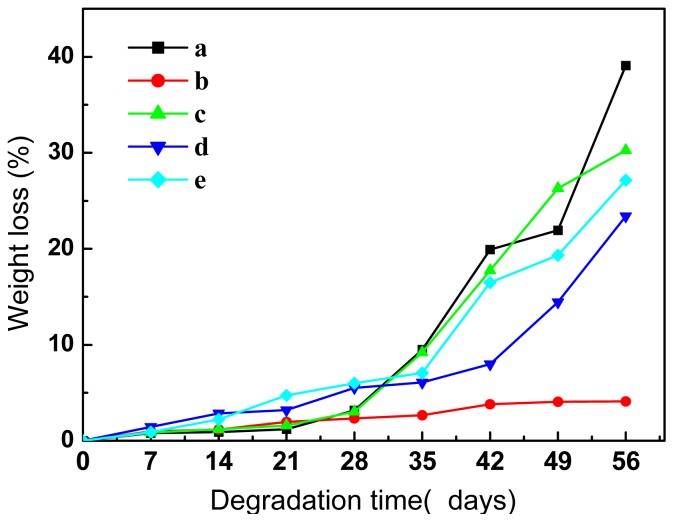
Weight loss of the examined samples as a function of degradation time: (**a**) PLA; (**b**) PBAT; (**c**) PLA/PBAT (80/20, *w*/*w*); (**d**) PLA/PBAT/ADR (80/20/1, *w*/*w*) and (**e**) PLA/PBAT/HDE (80/20/1, *w*/*w*).

The weight loss of PLA, PBAT, and PLA/PBAT blends are shown in Figure 6. The weight loss of neat PLA is approximately 3% in the initial stage, while it increases steeply after four weeks (Figure 6a). On the other hand, the weight of the PBAT sample is relatively stable during the experimental time (Figure 6b). The trend of weight loss of the PLA matrix was not changed by addition of 20 wt % of PBAT, however the rate of weight loss of the blend was slowed down by the addition of chain-extenders (Figure 6d,e). The lower weight loss rate is resulted from the higher molecular weight of the chain-extended samples.

#### 2.5.2. Molecular Weight Variation

The transition points of weight loss curves for most of the samples occur at around 35 days (Figure 6), thus the weight average molecular weights (*M̄*_w_ ) of the samples were measured at 0, 35, and 56 days respectively, and hydrolysis in the samples at different time interval was calculated. The results of molecular weight analysis are summarized in Table 3.

The *M̄*_w_ is used to evaluate the hydrolysis process since it is less affected by low *M*_w_ composition than number average molecular weight [20]. It has to be mentioned that bimodal distribution of the *M*_w_ was detected for some of the degraded samples, as listed in Table 3. The *M̄*_w_ of PLA is 150 kDa before degradation which is decreased to 14 kDa (39%) and 5 kDa (61%) after five weeks and further reduced to 10 kDa (9%) and 4 kDa (91%) after eight weeks. On the other hand, PBAT exhibits much better resistance to the hydrolysis, which is in accordance with the above discussion. The *M*_w_ of the samples dropped significantly within 35 days (by a factor of >5) except the PBAT indicating an obvious hydrolysis of the PLA in this period. Taking the composition of the PLA/PBAT blends (*i.e.*, 80/20, *w*/*w*) and the hydrolytic characters of the neat PLA and PBAT into account, it can be concluded from Table 3 that addition of the PBAT and chain-extenders did not obviously retard the hydrolysis of the PLA matrix while the PLA in the chain-extended blends showed higher *M̄*_w_ in the first few weeks. However the *M̄*_w_ of the PLA in different samples are similar after eight weeks.

#### 2.5.3. pH Variation

**Figure 7:**
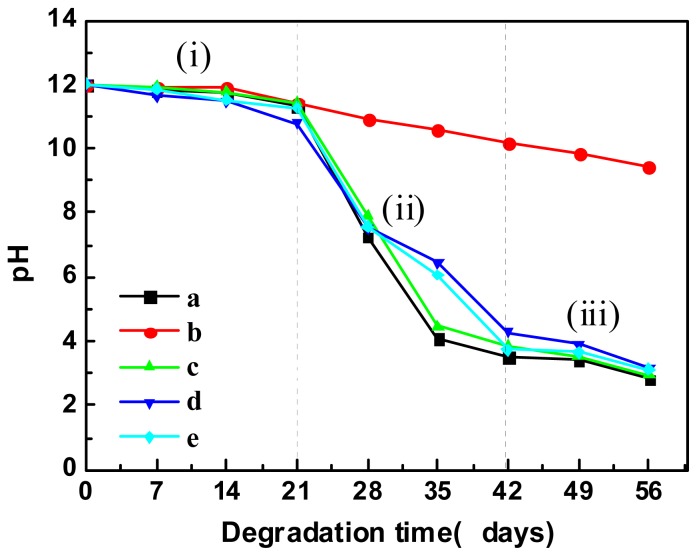
pH values of the hydrolytic mediums of (**a**) PLA; (**b**) PBAT; (**c**) PLA/PBAT (80/20, *w*/*w*); (**d**) PLA/PBAT/ADR (80/20/1, *w*/*w*); and (**e**) PLA/PBAT/HDE (80/20/1, *w*/*w*).

Carboxyl group (−COOH) and hydroxyl group (−OH) are generated after hydrolytic degradation, which decreases the pH values of the medium, as shown in Figure 7. The pH values of the mediums of the PLA and its blends slightly decreased in the first three weeks but dropped steeply (from 11 to 4) during the four to six weeks, after which the pH values leveled off in the range of 3–4. This pH range is coherent with the p*K*a of lactic acid oligomers [35]. Therefore, the hydrolysis of PLA was mainly occurred during the period of four to six weeks. On the other hand, the pH value of the medium of the PBAT was just decreased from 12 to 10 in eight weeks.

Water was assumed in literature to be uniformly distributed within the polyester from the beginning of erosion process, and hydrolysis promotes homogenous bulk erosion [36]. However, the trend of weight and pH variations of the samples (Figures 6 and 7) and the bimodal distribution of the molecular weight after degradation (Table 3) indicate heterogeneous bulk erosion as a function of time. Three stages of the hydrolytic process for the studied PLA samples are proposed (see Figure 7) based on the above discussion: (i) Gradual diffusion of water into the samples accompanied by slight hydrolysis from the surface to the bulk; (ii) Fast hydrolysis of the bulk leading to a fragility of the samples; and (iii) Further hydrolysis of the low-*M*_w_ PLA to be soluble lactic acid oligomers. The addition of chain-extenders modified part of the end groups of the polyesters while kept the ester bonds unchanged. Consequently, neither the hydrolytic trend nor the hydrolytic mechanism of the samples is influenced obviously by the chain-extenders.

## 3. Experimental Section

### 3.1. Materials

Poly(lactide) (PLA 2002D) with a melt flow index (MFI) of 7.0 g/10 min (210 °C/2.16 kg) was supplied by Natureworks LLC., Blair, NE. USA. Poly(butylene adipate-*co*-terephthalate) (PBAT) with a melt flow index of around 4.0 g/10 min (210 °C/2.16 kg) was supplied by Zhejiang Hangzhou XinFu Pharmaceutical Co., Ltd. (Hangzhou, China). Joncryl ADR-4370S abbreviated as ADR in this paper was supplied by BASF Corporation Shanghai China. 1,6-hexanediol diglycidyl ether abbreviated as HDE in this paper was provided by Wuxi Kaimike Electronic Materials Co., Ltd. (Wuxi, China). All the samples are used as received.

### 3.2. Blend Preparation

Both PLA and PBAT were dried in a vacuum oven at 50 °C for 12 h before use. The PLA/PBAT blends with different types and different amounts of chain-extenders were prepared in a mixing chamber of RM-200 Hapro Rheometer (Hapro Electric Manufacturing Co. Ltd., Harbin, China) at 175 °C and 50 rpm for 6 min. For comparison neat PLA and PBAT were also processed under the same conditions. After preheating, the samples were compression-molded into sheets at 190 °C and 10 MPa for 5 min using a hot compression molding machine. The compression-molded samples were used for further testing and characterization. Unless specified otherwise all the blending ratio mentioned in this paper refers to weight ratio.

### 3.3. Characterization

#### 3.3.1. Tensile Properties

The tensile properties of the samples were tested by using a KD111-5 tensile tester (Shenzhen KQL testing instruments Co. Ltd., Shenzhen, China) at a crosshead speed of 5 mm/min according to GB/T 1040.3-2006 standard. The dumbbell-shaped specimens for tensile testing were stamped from the compression-molded sheets with a dimension of 33 mm in length, 0.5 mm in thickness and 6 mm in width of the parallel portion. All the tests were performed at room temperature.

#### 3.3.2. Scanning Electron Microscopy (SEM)

SEM (S-4800, Hitachi, Ibaraki, Japan) was used to characterize the phase morphology of the PLA/PBAT blends with and without chain-extenders and the eroded morphology of the degraded samples. The surfaces of the samples were observed by using the SEM after sputter-coating with a thin gold layer.

#### 3.3.3. Gel Permeation Chromatography (GPC)

The weight average molecular weight (*M̄*_w_ ) and molecular weight distribution (PDI) of the samples were measured by using GPC (Waters 1515, Milford, MA, USA), HPLC columns (styragel^®^ HR4, THF, 7.8 mm × 300 mm) and Waters 2414 refractive index detector. Mono-dispersed poly(styrene) was used as calibration standards. Solutions of the PLA, PBAT, and PLA/PBAT blends were prepared as 5–10 mg/mL in THF. The flow rate is 1.0 mL/min.

#### 3.3.4. Differential Scanning Calorimeter (DSC)

Thermal properties of the samples were measured by using DSC (Mettler Toledo 822e, Zurich, Switzerland). Around 5 mg of each sample was first heated from 30 to 200 °C and held for 5 min at 200 °C to erase its thermal history. The samples were then cooled to 30 °C and reheated to 200 °C. The measurements were performed in a nitrogen atmosphere with a scanning rate of 10 °C/min. The thermal parameters such as glass transition temperature (*T*_g_), cold crystallization temperature (*T*_cc_), melting point (*T*_m_), and melt enthalpy (*ΔH*_m_) were obtained from the second DSC heating runs.

#### 3.3.5. Hydrolytic Degradation Behavior

Small pieces of the samples with equal size and thickness (0.5 mm) were tailored for hydrolytic degradation testing. Nine pieces of each sample were sealed in nine reagent bottles respectively. Each bottle was filled with the same volume of 0.01 mol/L NaOH standard solution. To follow the hydrolytic degradation in a reasonable time range, the experiments were performed at 60 °C in alkaline medium. All the samples were dried and weighted (*m*_0_) before the experiments. Then the samples were taken out and washed with distilled water at different time (interval = 7 days). The washed samples were weighed (*m_i_*) after drying in a vacuum oven at 40 °C for 12 h. The weight loss (*W*_L_) of the samples was calculated via Equation (1):

(1) $$W_{\text{L}}=(m_{0}-m_{i})/m_{0}\times 100\%$$

where *m*_0_ is the weight of the samples before degradation and *m_i_* is the dry weight of the degraded samples. Meanwhile the molecular weight (*M*_w_) of the samples and the pH of the degradation medium were followed as a function of time. The *M*_w_ was measured by using the GPC (as described in section 3.3.3) and the pH was measured via a PHS-25 pH meter (Shengci Instrument Co. Ltd., Shanghai, China) at 25 °C. Furthermore, the eroded morphology of the samples after degradation was observed by using SEM.

## 4. Conclusions

Bio-based and biodegradable poly(lactide) (PLA) is an aliphatic polyester with notable brittleness. The ductility of PLA can be improved to a certain extent by incorporation of poly(butylene adipate-*co*-terephthalate) (PBAT). However the compatibility between the PLA and PBAT is poor leading to coarse morphology and limited elongation at break of the blends. Two chain-extenders with multi-epoxy groups were introduced to the PLA/PBAT blends to improve the compatibility and the properties of the blends. The chain-extending effect was confirmed by an increase in molecular weight (*M*_w_). A small amount of the chain-extenders increased the compatibility of the blends obviously as evidenced by a reduction of the PBAT domain size and an enhancement in the interfacial adhesion. Meanwhile the elongation at break of the PLA/PBAT blends was increased from 200% to around 500% without compromising the tensile strength. The crystallization rate of the PLA was decreased by the PBAT but was sped up after the chain-extension due to the heterogeneous nucleation effect of the compatibilized PBAT domains. The hydrolytic degradations of the samples were evaluated by following the weight loss of the samples, *M*_w_ reduction of the samples and the pH variation of the degradation medium as a function of time. The hydrolysis of PLA and its blends go through a gradual diffusion of water into the samples, fast hydrolysis of the bulk materials leading to a steep reduction in *M*_w_ and a further hydrolysis of the low-*M*_w_ PLA to be soluble lactic acid oligomers. The degradation trend/behavior of the PLA was not affected so much by the PBAT and the chain-extenders.

## Figures and Tables

**Table 1:** Molecular weight analyses of the samples measured via gel permeation chromatography.

Samples	*M̄*_n_ (kDa)	*M̄*_w_ (kDa)	PDI a
Neat PLA	90	150	1.7
Neat PBAT	30	65	2.2
PLA/PBAT (80/20, *w*/*w*)	60	130	2.2
PLA/PBAT/ADR (80/20/1, *w*/*w*)	100	330	3.3
PLA/PBAT/HDE (80/20/1, *w*/*w*)	70	160	2.3

a Molecular weight distribution index calculated via MDI = *M̄*_w_/*M̄*_n_ (MDI or PDI); *M̄*_n_ represents the number average molecular weight while *M̄*_w_ represents the weight average molecular weight.

**Table 2:** Thermal properties of the PLA and PLA/PBAT blends with different types of chain extenders.

Sample	*T*_g_ (°C)	*T*_cc_ (°C)	*T*_m_ (°C)	*ΔH*_m_ (J/g) b
PLA	65	128	156	21
PLA/PBAT (80/20, *w*/*w*)	64	130	155	18
PLA/PBAT/ADR (80/20/1, *w*/*w*)	63	123	152/157 a	28
PLA/PBAT/HDE (80/20/1, *w*/*w*)	61	123	152/156 a	23

a Double-melting peaks;

b The melt enthalpy of the PLA phase, which is apparent melt enthalpy of the blend divided by 80%.

**Table 3:** Molecular weight analysis of the samples at different degradation time.

Samples	Parameters	Days of degradation		

		0	35	56
PLA	*M̄*_w_ (kDa)	150	14/5	10/4
	PDI	1.7	1.4/0.5	1.0/1.1
	ratio (%)	100	39/61	9/91

PBAT	*M̄*_w_ (kDa)	65	42	27/1
	PDI	2.2	2.1	2.7/1.0
	ratio (%)	100	100	97/3

PLA/PBAT	*M̄*_w_ (kDa)	130	15/5	13/4
	PDI	2.2	1.2/1.1	1.2/1.2
	ratio (%)	100	54/46	27/73

PLA/PBAT/ADR (80/20/1, *w*/*w*)	*M̄*_w_ (kDa)	330	67	13/5
	PDI	3.3	3.1	1.1/1.1
	ratio (%)	100	100	26/74

PLA/PBAT/HDE (80/20/1, *w*/*w*)	*M̄*_w_ (kDa)	160	20/2	12/4
	PDI	2.3	1.8/1.0	1.1/1.2
	ratio (%)	100	97/3	28/72

Two values in the same table cell (e.g., *x*/*y*) are due to bimodal distribution of molecular weight. *M̄*_w_ is weight average molecular weight and PDI represents the molecular weight distribution. The percentage of each component with a corresponding *M̄*_w_ is listed in the row of “Ratio”.
